# Age Does Not Predict Mortality in Hospitalized COVID-19+ Older Adults: Rethinking Resource Allocation Based on Age

**DOI:** 10.1093/geroni/igaa057.3419

**Published:** 2020-12-16

**Authors:** Liron Sinvani, Allison Marziliano, Alex Makhnevich, Meng Zhang, Maria Carney, Michael Diefenbach, Karina Davidson, Edith Burns

**Affiliations:** 1 Zucker School of Medicine at Hofstra/Northwell, Manhasset, New York, United States; 2 Feinstein Institute, Hofstra Northwell School of Medicine, Manhasset, New York, United States; 3 Northwell Health, New Hyde Park, New York, United States; 4 Northwell Health, Manhassett, New York, United States; 5 Northwell Health, New York, New York, United States

## Abstract

Older adults are disproportionately affected by the coronavirus (COVID-19) pandemic. While age has been used to guide resource allocation based on studies implicating age as the main risk factor for COVID-19-related mortality, most did not account for critical factors such as baseline functional and cognitive status, or life-sustaining treatment preferences. The objective of this study was to determine whether age is independently associated with mortality in older adults hospitalized with COVID-19. We conducted a retrospective cohort study of adults age 65+ with confirmed COVID-19 hospitalized in the greater NY metropolitan area between 3/1/20-4/20/20. Primary outcome was in-hospital mortality, with age as the primary predictor. Multivariate logistic regression was used to evaluate association between age and in-hospital mortality after controlling for demographics, severity of acute illness, comorbidities, and baseline function, cognition, and life-sustaining treatment preferences. 4,969 patients were included, average age 77.3, 56.0% male, 46.8% White, 20.8% African American, 15.1% Hispanic. Common comorbidities included hypertension (61.1%), and diabetes (36.8%); average number of comorbidities was 3.4 (SD 2.8) and 13.0% had dementia. 20.8% arrived from a facility and 5.7% had early do-not-resuscitate orders. On arrival, the Modified Early Warning System score was 4.2 (SD 1.7) and 79.6% required oxygen therapy. 35.3% of patients expired. In multivariate analysis, age was not independently associated with mortality (p = .173). Functional status, multi-morbidity, life-sustaining treatment preferences, and illness severity, not age, were associated with mortality among older adults hospitalized with COVID-19, suggesting age should not be used as the main indicator to guide resource allocation.

